# Factors influencing follow-up care post-TIA and minor stroke: a qualitative study using the theoretical domains framework

**DOI:** 10.1186/s12913-022-07607-0

**Published:** 2022-02-21

**Authors:** Grace M. Turner, Maria Raisa Jessica V. Aquino, Lou Atkins, Robbie Foy, Jonathan Mant, Melanie Calvert

**Affiliations:** 1 Institute of Applied Health Research, University of Birmingham, B15 2TT, Birmingham, UK; 2 Centre for Patient Reported Outcomes Research, University of Birmingham, B15 2TT, Birmingham, UK; 3 NIHR Surgical Reconstruction and Microbiology Research Centre, University Hospitals Birmingham NHS Foundation Trust and University of Birmingham, B15 2TH, Birmingham, UK; 4 Population Health Sciences Institute, Faculty of Medical Sciences, Newcastle University, Newcastle upon Tyne, NE1 7RU UK; 5 Primary Care Unit, Department of Public Health and Primary Care, University of Cambridge, Cambridge, CB1 8RN UK; 6 Centre for Behaviour Change, University College London, WC1E 6BT, London, UK; 7 Leeds Institute for Health Sciences, University of Leeds, Leeds, LS2 9JT UK; 8 NIHR Birmingham Biomedical Research Centre, University Hospitals Birmingham NHS Foundation Trust and University of Birmingham, B15 2TH, Birmingham, UK; 9 Birmingham Health Partners Centre for Regulatory Science and Innovation, University of Birmingham, B15 2TT, Birmingham, UK; 10 NIHR Applied Research Collaboration (ARC) West Midlands, University of Birmingham, B15 2TT, Birmingham, UK

**Keywords:** Transient ischaemic attack, TIA, Minor stroke, Follow-up, Theoretical domains framework

## Abstract

**Background:**

Follow-up care after transient ischaemic attack (TIA) and minor stroke has been found to be sub-optimal, with individuals often feeling abandoned. We aimed to explore factors influencing holistic follow-up care after TIA and minor stroke.

**Methods:**

Qualitative semi-structured interviews with 24 healthcare providers (HCPs): 5 stroke doctors, 4 nurses, 9 allied health professionals and 6 general practitioners. Participants were recruited from three TIA clinics, seven general practices and one community care trust in the West Midlands, England. Interview transcripts were deductively coded using the Theoretical Domains Framework and themes were generated from coded data.

**Results:**

There was no clear pathway for supporting people with TIA or minor stroke after rapid specialist review in hospital; consequently, these patients had limited access to HCPs from all settings (‘Environmental context and resources’). There was lack of understanding of potential needs post-TIA/minor stroke, in particular residual problems such as anxiety/fatigue (‘Knowledge’). Identification and management of needs was largely influenced by HCPs’ perceived role, professional training (‘Social professional role and identity’) and time constraints (‘Environmental context and resources’). Follow-up was often passive – with onerous on patients to seek support – and predominantly focused on acute medical management (‘Intentions’/‘Goal’).

**Conclusions:**

Follow-up care post-TIA/minor stroke is currently sub-optimal. Through identifying factors which influence follow-up, we can inform guidelines and practical strategies to improve holistic healthcare.

**Supplementary Information:**

The online version contains supplementary material available at 10.1186/s12913-022-07607-0.

## Introduction

Transient ischaemic attack (TIA) and minor stroke are important risk factors for major stroke. Over 46,000 people experience a first TIA or minor stroke per year and half a million people live with TIA and/or minor stroke in the United Kingdom [1].

National guidelines promote rapid diagnosis and long-term management that focuses on stroke prevention [2–4]. However, many people with TIA and minor stroke feel unsupported in stroke prevention – both medication and lifestyle change – and often lack basic understanding of their diagnosis, stroke risk and preventative medication [5]. Furthermore, many people experience important residual impairments post-TIA and minor stroke; including, fatigue, psychological and cognitive problems [5–9]. These impairments can affect quality of life, ability to return to work or usual activities, and relationships with family and friends [5, 10–15]. Therefore, person-centred care after TIA and minor stroke should be multifaceted and encompass (i) information provision (diagnosis and stroke risk); (ii) stroke prevention (medication and lifestyle change); and (iii) holistic needs (residual problems and return to work and usual activities) [5, 16]. However, follow-up care is reported to be inadequate and variable with patients feeling abandoned after specialist review [5].

Several studies have explored TIA and minor stroke patients’ perspectives of initial treatment and follow-up [10, 14, 16]; however, none have investigated healthcare providers (HCPs) experiences. To improve future care, it is important to identify gaps in follow-up healthcare provision and understand influences on related HCP behaviours. This qualitative study aimed to explore factors influencing holistic follow-up care post-TIA/ minor stroke among different HCPs (doctors, nurses, allied health professionals [AHPs]) and across different health care settings (primary, secondary and community care).

## Methods 

### Study design

Qualitative, theory-guided, semi-structured interviews. A qualitative approach was selected as this study focuses on exploring attitudes and perceptions of HCPs. Semi-structured interviews were preferred to focus groups because it allowed the researcher to obtain richer and deeper data.

### Theoretical domains framework

Changing clinical practice requires an understanding of the influences on behaviour; therefore, our study was underpinned by the Theoretical Domains Framework (TDF) [17]. This theoretical framework was developed for implementation research to identify influences on HCP behaviour [17]. We used the 14-domain TDF (v2) (see Additional file 1: Appendix eTable 1). The first stage of TDF-based research is to select and specify the target behaviour(s) [17]. In our study, target behaviours were (i) identification and (ii) management of individuals’ needs post-TIA/ minor stroke. ‘Needs’ were informed by the literature and defined as: information provision (diagnosis and stroke risk); supporting stroke prevention (medication and lifestyle change); and addressing holistic needs (residual problems and return to work or usual activities).

### Participants

Participants were HCPs who work with TIA or minor stroke patients, including: secondary care doctors, nurses or AHPs; primary care general practitioners [GPs]; and community care AHPs and nurses. Convenience and snowball sampling were used initially; however, sampling became increasingly purposeful to achieve variation in clinical role (doctor, nurse, AHP) and setting (primary, secondary, community care). Secondary care doctors, nurses and AHPs were recruited from three TIA clinics in the West Midlands (England). Community HCPs were recruited from Birmingham Community Healthcare Trust. GPs were recruited from two general practices in the West Midlands and through snowballing. A pragmatic approach to site selection was used whereby research invitations were sent to potential sites and those that responded first were selected. Recruitment rates (percentage of HCPs interviewed of those invited to participate) were: 86% (12/16) for secondary care, 60% (6/10) for community care and 43% (6/14) for GPs.

### Data collection

One-to-one, semi-structured interviews were conducted by telephone or face-to-face (at the University of Birmingham or participants’ workplace). All interviews were conducted by GT, a female, non-medical researcher trained in qualitative research methods. All participants were interviewed once. The interviewer did not have a relationship with any of the participants.

Topic guides were informed by existing literature and consultation with the research team and patient partners, and refined through piloting with patient partners. Topic guides covered: follow-up pathways and communication between healthcare settings, and current practice to identify and address needs. Participants also completed a short demographic questionnaire. Fieldnotes were taken during and after each interview to add contextual information, and reflect on how the interview was conducted. Adjustments were made if necessary during the following interviews, thereby improving reflexivity and trustworthiness of the data.

Digital audio recorded interviews were transcribed verbatim by a professional transcription service. Interviews were conducted between March and November 2018, until the research team judged that the sample and data had sufficient depth and breadth to address the research questions.

### Analysis

Computer Aided Qualitative Data Analysis Software (CAQDAS) QSR NVivo 12 supported the sorting, coding and organisation of transcribed data. A key advantage of using CAQDAS software is that it facilitates and enhances researcher reflexivity [18]. Transcripts were read several times to enable familiarisation with the interviews. Participant responses were deductively coded into relevant TDF domains. GT coded all transcripts and RA independently coded a subset (10%, *n* = 2). GT and RA are both experienced qualitative researchers. Responses coded in different domains were discussed by GT and RA to establish a consensus; all differences in coding were resolved. Themes were iteratively generated from coded data. Initial data coding and generation of themes was concurrent with the interviews and data collection stopped when no new themes were identified. Themes were identified initially by GT, reviewed by RA, and developed and refined by discussion between GT and RA. The wider research team and our patient partners met on a regular basis to discuss and further refine the themes.

### Ethical approval and participants’ informed consent

Ethical approval was given by the Warwickshire North West - Greater Manchester East Research Ethics Committee (Reference: 17/NW/0737). Written or recorded verbal informed consent was obtained from the participants, by the interviewer, immediately prior to the interview. All methods were carried out in accordance with the UK Policy Framework for Health and Social Care Research and the Declaration of Helsinki.

## Results

The final sample consisted of 24 HCPs (5 stroke doctors, 4 nurses, 9 AHPs and 6 GPs). Participants’ years of experience ranged from 3 to 37 (mean 15, standard deviation 9), characteristics are summarised in Table 1 and detailed in eTable 2 (see Additional file 1: Appendix). Mean interview length was 48 min (range 34 to 68 min).

**Table 1:** Characteristics of participants (*n* = 24)

Variable		Number (%)
**Age (years)**	21–30	1 (4.2)
	31–40	8 (33.3)
	41–50	12 (50.0)
	51–60	3 (12.5)
**Sex**	Male	10 (41.7)
	Female	14 (58.3)
**Profession**	Stroke consultant^a^	5 (20.8)
	Nurse	4 (16.7)
	Allied health professional	9 (37.5)
	General practitioner	6 (25.0)
**Healthcare setting**	Secondary care	9 (37.5)
	Primary care	6 (25.0)
	Community care	6 (25.0)
	Secondary & community care	3 (12.5)
**Years of experience**	< 5	3 (12.5)
	5–10	5 (20.8)
	11–20	11 (45.8)
	> 20	5 (20.8)

^a^ physicians with specialist skills in stroke medicine

Eleven TDF domains were identified as relevant: Environmental context and resources; Social influences; Knowledge; Intentions; Memory, attention and decision processes; Social/professional role and identity; Beliefs about capabilities; Beliefs about Consequences; Reinforcement; Skills; and Goals (Tables 2 and 4).

**Table 2:** Theoretical domains and sample quotes related to pathways and access to follow-up care

TDF domain	Theme (Barrier [B], Enabler [E], Mixed [M])	Quote
**Lack of standardised follow-up care pathway**		
Environmental context and resources	Variability in follow-up pathways (M)	*“So we found that with some TIA clinics they offer a follow up appointment around six to eight weeks, sometimes it’s consultant led and sometimes it’s nurse led so you can imagine that those appointments would be very different depending on who they speak to whereas other TIA clinics don’t have that option at all so there’s a very disjointed follow up pathway which people are getting...”* [H8, GP, 17 Years of experience]
	Restricted access to early supported discharge (B)	*“So, for the TIAs obviously we don’t do that* [Early Supported Discharge] *there’s no follow up.”* [H17, Stroke consultant, 8 Years of experience]
Intentions	Variability in consultants use of nurse-led follow-up (M)	*“We do have a, for follow-up we do have a nurse lead follow-up clinic. Which I have access to, but I don’t use a lot. And again, there’s some variation in practice amongst the five stroke physicians about how much they use that clinic.”* [H20, Neurologist, 22 Years of experience]
	Variability in GPs having an active vs passive approach to follow-up (M)	*“…personally I quite like to see patients particularly when patients have been started on a whole bunch of new tablets… So, I like to get them to come and see me.”* [H11, GP, 18 Years of experience] *“…it wouldn’t be feasible for every specialist letter we get for strokes and everything else to contact the patient to sort of go through the* [medication]*, we wouldn’t do anything else really. So we add the medication to the repeat prescription”* [H13, GP, 13 Years of experience]
**Interface between healthcare settings**		
Environmental context and resources	Restricted communication between healthcare settings (B)	*“So, I think for me part of the problem is sometimes the access to the specialist. And yeah, we can fax over letters and we can make phone calls and we can try and bleep people and we can email and all this sort of thing, but you know my experience generally is that we don’t get a lot of information back.”* [H11, GP, 18 Years of experience]
	Variability in content and speed of discharge letters (M)	*“Communication is quite good. It’s quick. The turnaround on letters is quick…”* [H3, AHP, 23 Years of experience] *“We’re still relying on old paper letters, which, you know, we probably shouldn’t be anymore and communication is very slow, so it takes us six weeks often to get a clinic letter and if something’s urgent then we can’t afford to wait that long.”* [H13, GP, 13 Years of experience]
Intentions	Variability in how GPs engage with discharge letters (M)	*“And we tend to just wait for the* [discharge] *letter, act on it… It’s very much been directed by the secondary care rather than doing a massive amount off our own backs.”* [H12, GP, 7 Years of experience] *“And certainly the discharge letters are quite ‘protocolised’ again in that… And there’s things that they will put on there that you just think ‘well, there’s no need for that to be on there’ in terms of giving me advice here…”* [H11, GP, 18 Years of experience]
Social Influences	GPs have difficulty accessing imaging results and specialist stroke advice (B)	*“But obviously it’s a bit hard to give them* [patients] *absolute reassurance because in terms of the scan reports or the results on the investigations, may not be entirely with us…”* [H9, GP, 6 Years of experience]

There were 3 overarching themes and 10 sub-themes:

1. Pathways and access to follow-up care (Lack of standardised follow-up care pathway; Interface between healthcare settings; quotes are provided in Table 2).
2. Identifying needs (Approach to identifying needs; Use of checklists and screening tools; Patient factors; quotes are provided in Table 3)
3. Addressing needs (Stroke prevention; Residual impairments; Education about diagnosis, stroke risk and medication; Use of support services and resources; Patient factors; quotes are provided in Table 4)

**Table 3:** Theoretical domains and sample quotes related to identifying needs

TDF domain	Theme (Barrier [B], Enabler [E], Mixed [M])	Quote
**Approaches to identifying needs**		
Social professional role and identity	Perceived role in follow-up care influences approaches to identifying needs (M)	*“I: So, do you see that a part of your role to ask about things like people’s social activities and their mood and cognition and the more holistic side?* *IV: Yeah, I think it is part of our role…”* [H22, nurse, 13 Years of experience] *“Usually it’s pretty much a one-stop clinic so if they need a carotid scan they get it there. If they need a brain scan urgently they get it there. We give them the medication that they need to prevent further events, book any other tests which are non-urgent but still need to be done and then we discharge them. So it’s a one-stop medical clinic.”* [H24, consultant, 12 Years of experience]
	Professional training influenced approaches to identifying needs (M)	*“As an OT, obviously we’re dual trained in physical and mental health.”* [H14, AHP, 17 Years of experience]
Knowledge	Knowledge/ lack of knowledge of potential patient needs (M)	*“…but there might be a lack of education, medical education about the, yeah, the long-term consequences really.”* [H13, GP, 13 Years of experience] *“…and that can affect you, you know you can’t drive, you maybe can’t work, can’t watch TV, can’t read, it’s a very small minor stroke but it’s had a big effect.”* [H4, nurse, 37 Years of experience]
Goal	HCPs had different perceptions on the goal of their follow-up (M)	*“In the review clinic, we make sure two things, one, that all investigations have been completed. Secondly all the risk factors have been addressed and thirdly they’re on the right medications for the conditions. So, we just see them one more time after being seen in the TIA clinic.”* [H7, consultant, 20 Years of experience] “*And it’s quite a holistic type clinic so we look at them although obviously we’re focussing on the stroke, we’re looking at the whole person.*” [H4, nurse, 37 Years of experience] “*…so the follow up that I offer tends to be just checking that they’re ok, that they’re sort of getting on with their medications that they have recently been prescribed and just ensuring that they are kind of informed about you know what the process is and any further results that are coming back through and I guess sort of just general support about you know ongoing risk factors and risk reduction…”* [H8, GP, 17 Years of experience]
Intentions	Active vs passive approach to identifying needs (M)	*“I don’t actively ask for it, I don’t actively for sleep and emotional problems, not things that I tend to ask about…”* [H20, consultant, 22 Years of experience] *“So, there’s an element of tailoring. But we do always generally check mood, fatigue, confidence as well, as part of what we’re doing.”* [H22, nurse, 13 Years of experience]
Social influences	Personal experience of TIA/minor stroke (E)	*“…but it almost seems like there is a kind of post TIA syndrome and certainly I probably first became aware of that through personal experience really rather than in the practice.”* [H8, GP, 17 Years of experience]
Beliefs about capabilities	Confident/ not confident in identifying needs (M)	*“I: Do you feel quite confident in being able to identify what their [patients’] needs are?* *IV: Yeah. yeah, I think I definitely…”* [H16, AHP, 4 Years of experience]
**Use of checklists and screening tools**		
Environmental context and resources	Checklists/ screening tools used/ not used to facilitate identification of needs (M)	*“We use formal mood screens in [location]… the HADS, the DISC, those types of things. In terms of fatigue we use self-rating scales for fatigue. Obviously, cognition we’ve got a whole host of standardised assessments that we use, alongside functional assessments as well. Anxiety again, would be self-rating. And fear of falling would be self-rated. We don’t use every single one with every single patient but we have those.”* [H14, AHP, 17 Years of experience] *We don’t, at the moment, do a formal mood assessment and we don’t do a formal cognitive screen within clinic.”* [H3, AHP, 23 Years of experience]
	Lack of time to use screening tools (B)	*“…in a clinic setting, there isn’t really time to do lots of formal screening.”* [H3, AHP, 23 Years of experience]
Beliefs about consequences	Checklist/ screening tools considered useful/ not useful (M)	*“I mean the screen tools don’t always pick up on these things and sometimes we’ve found that, you know, at home they answer that everything’s alright on the PHQ’s but actually when you see them they are clearly upset about something.”* [H8, GP, 17 Years of experience] *“It [checklist] just gets a good, overall idea of what they’re doing and then identifies then at the end of it what they need to be referred to.”* [H1, AHP, 5 Years of experience]
Reinforcement	Content of primary care long-term conditions template is influenced by performance-based incentives (Quality and Outcomes Framework) (B)	*“Cause one of the things we have at present in our clinical systems is templates… I think they tend to be very much QOF kind of based. So, it’ll probably be addressing things like cholesterol, blood pressure, their sugar* etc. etc. *Medication, making sure they are on the appropriate medications… I don’t think it actually addresses the kind of psychological aspects.”* [H10, GP, 31 Years of experience]
	Screening tool mandated by local Clinical Commissioning Group (B)	“…the Barthel Index is obviously the Clinician Commissioning Group level, so I don’t think that will change…” [H2, AHP, 3 Years of experience]
Memory, attention and decision processes	Checklist/ screening tool used to inform decision making (E)	*“…then we’ll use it* [screening tool] *to set the goals and then we’re doing it to give them to focus. What we want to do is improve their score and also so that we can monitor that what we’re doing is effective as well.”* [H5, AHP, 16 Years of experience]
Skill	Skilled/ not skilled in use and interpretation of screening tools (M)	*“…we’ve trained the physios to do MOCAs, so, we provide weekend service on the wards. Unfortunately not in ESD at the moment, so, if a physio is working at the weekend, they can do a MOCHA over the weekend, so, there isn’t that delay.”* [H14, AHP, 17 Years of experience]
**Patient factors**		
Social influences	Cultural/ language barriers (B)	*“The obvious one is language and non-English speaking patients where you may not know that until they come to clinic and you’re really then stuck…”* [H3, AHP, 23 Years of experience]
	Patients not wanting to “bother” doctor or raise non-medical issues (B)	*“…especially elderly people they don’t pester their GP for things, my mum says that, I don’t want to trouble the GP.”* [H4, nurse, 37 Years of experience]
	Family members as facilitators/ barriers to identification of patient needs (M)	*“The other one is just normally again partners coming in and it tends to be men who come in and they don’t say a great deal and then the partner or wife mentions they’re worried that the patient’s been like this for a long time and then they tell me everything.”* [H12, GP, 7 Years of experience] *“…people can be quite proud and not want to sort of, they want to put a good front on it for other family members and things and not admit it.”* [H6, AHP]

**Table 4:** Theoretical domains and sample quotes related to addressing needs

TDF domain	Theme (Barrier [B], Enabler [E], Mixed [M])	Quote
**Stroke prevention**		
Social professional role and identity	HCP did/ did not perceive prescribing stroke prevention their role (M)	*“I: Do you see that as part of your role as well, around the stroke prevention, diet and exercise?* *IV: Totally. So, in terms of stroke prevention, we do lots of education with our patients that come through to ESD.”* [H14, AHP, 17 Years of experience] *“I think for TIAs it would be more kind of about prevention, wouldn’t it, really. Lifestyle and education and prevention, which wouldn’t really be our role.”* [H5, AHP, 16 Years of experience]
Belief about capabilities	Confident/ not confident in prescribing stroke prevention medication (M)	*“…But certainly, blood pressure always worries me, I don’t think we treat blood pressure well. I don’t treat it, I don’t manage it because I’m a Neurologist by training. So, I think I lack expertise…”* [H20, Neurologist, 22 Years of experience]
Environmental context and resources	Lack of time to address lifestyle change (B)	*“So, we talk about stopping smoking and healthy diet and exercise but it’s a fairly brief discussion and don’t really feel I have time in the clinic to do that in great depth.”* [H20, consultant, 22 Years of experience]
	Information leaflets used to address lifestyle change (E)	*“Again, we use a lot of the Stroke Association’s resources, like leaflets about exercise after stroke, prevention and risk of stroke which we tend to give out to patients.”* [H1, AHP, 5 Years of experience]
Intentions	GPs actively reviewed patients’ medication vs issuing repeat prescriptions from secondary care (M)	*“Usually what would happen is we get a letter from the specialist and we add the medication that they’ve suggested onto the person’s repeat medications.”* [H13, GP, 13 Years of experience] *“…personally I quite like to see patients particularly when patients have been started on a whole bunch of new tablets… So, I like to get them to come and see me.”* [H11, GP, 18 Years of experience]
	Lifestyle change not meaningfully addressed or actively supported (B)	“*Diet maybe we could improve, I don’t talk a lot about diet I’ll just say generally healthy diet and that’s all I’ll say. Smoking, I’ll tell people to stop smoking but I won’t talk about medication for that…”* [H20, consultant, 22 Years of experience]
Goal	Stroke prevention was/ was not considered a goal of HCPs’ follow-up (M)	*“The whole point [of a TIA clinic] is that they’re at increased risk early, so the whole point to come and see them early is to get treatment started early.”* [H21, consultant, 24 Years of experience]
Beliefs about consequences	Lifestyle change considered important for stroke prevention (E)	*“I’m very passionate about how lifestyle can change your life or has an effect on your life.”* [H17, consultant, 8 Years of experience]
**Residual impairments**		
Knowledge	Knowledge/ lack of knowledge of residual impairments (M)	*“I don’t think there is an awareness that there are these long-term sequalae... So there’s probably a bit of a lack of, well speaking personally, I don’t know what my colleagues would say, but there might be a lack of education, medical education about the, yeah, the long-term consequences really.”* [H13, GP, 13 Years of experience] *“But actually, from our experience, we see a lot of patients who have had a TIA and you know, they may only have had symptoms for 10 min but actually, there are a lot of other, what we call sort of hidden effects that I think are missed.”* [H22, nurse, 13 Years of experience]
Beliefs about capabilities	Confidence/ lack of confidence in addressing residual problems (M)	*“I don’t think I will bring back somebody to manage their mood and fatigue because I don’t feel competent in doing that and probably I’m not.”* [H17, consultant, 8 Years of experience] *“Yeah, I feel confident being able to then draw from that whether they needed directing to further psychology referral or whether it’s maybe just providing that sort of information with regards to prevention or what they’ve actually been through. I’d feel quite confident being able to do that, from having talked to them.”* [H1, AHP, 5 Years of experience]
Intentions	Stroke prevention prioritised over residual problems (B)	*“I guess I prioritise things which I think are extremely important, so smoking advice, cessation advice, exercise, restrictions into what they can do... The things which are absolutely mandatory to make sure that they completely understand the importance of their medication and why they’re taking it and what side effects they may get, that sort of thing; things which are commonly going to arise. I guess I probably don’t spend so much time, unless they specifically ask, about the slightly more quality of life activities of living questions that they may have.”* [H24, consultant, 12 Years of experience]
Skill	Some AHPs/nurses had the skills to actively addressed residual needs (E)	*“As an OT, obviously we’re dual trained in physical and mental health. So, we do have a certain basic training in terms of anxiety management skills, anger management, those types of things.”* [H14, AHP, 17 Years of experience]
Beliefs about consequences	AHPs/ nurses believed in the value of a “supportive chat” which involved active listening, acknowledging patients’ needs and reassurance (E)	*“But then a lot of the time spent with them doing the supportive chat will be reassurance wouldn’t it you know. I think a lot of people I think supportive chat and reassurances is a big thing that people perhaps require and then if they don’t have that that’s when things build up and the stress levels and stuff are worse.”* [H4, nurse, 37 Years of experience]
**Education about diagnosis, stroke risk and medication**		
Intentions	HCP provided/ did not provide education (M)	*“Rightly or wrongly I think we have to really make the assumption that the patient has been counselled adequately about that medication and why they’re being put on it in secondary care, yes, because otherwise it’s duplication of work”* [H13, GP, 13 Years of experience] *“We do a lot of education on exercise and basically, the benefits of keeping active, diet, alcohol and smoking.”* [H1, AHP, 5 Years of experience]
Beliefs about consequences	Belief that it is difficult for patients to retain information provided at the acute stage (M)	*“in the acute phase when patients are seen [patients get] an awful lot of information ... And so the amount of information that they absorb is tricky...”* [H11, GP, 18 Years of experience]
**Use of support services and resources**		
Environmental context and resources	HCPs used/ did not use support services (M)	*“Smoking cessation I have to say I don’t often refer them to a smoking cessation clinic. I’m guilty of not doing that…”* [H24, consultant, 12 Years of experience] *“The main ones that we do refer to tend to be the Stroke Association... There’s a Recover group, I think, for alcohol and drug substance misuse. We’ve got referral forms for quite a different few and various links for different reasons and we would refer if necessary.”* [H1, AHP, 5 Years of experience]
	Lack of support services (B)	*“The other side of the problem is that there is very little to refer to.”* [H3, AHP, 23 Years of experience]
	Barriers to accessing support services, including long wait times, referral processes, transport issues and geographical boundaries (B)	*“I mean at the moment, again, it’s the waiting times, a lot of people complaining, that I’ve been told, I’ve rung them, but they see they can’t see me for eight weeks, ten weeks, something like that.”* [H7, consultant, 20 Years of experience] *“I always feel like there are big geographical gaps.* [H3, AHP, 23 Years of experience] *“I think transport is a huge issue.”* [H3, AHP, 23 Years of experience]
	Directories used to facilitate identification of support services (E); however, these were often outdated (B). Successful directories had someone delegated to update them (E)	*“We have our own directory … and basically what people do, what’s the name, how do you refer and how do you access. We already have an inhouse directory... We have an admin person that manages that directory, any new updates, any new differences to the referral pathway, any different forms, that is updated by our admin staff…”* [H9, GP, 6 Years of experience] *“…but I must admit in the past when I’ve been sent little directories of support services, they are useful but it suddenly becomes limited after about six or twelve months because a lot of these organisations are they don’t sustain… they just change or they move or whatever…”* [H8, GP, 17 Years of experience]
	In primary care, access to social prescribers or community champions facilitated identification of support service (E)	*“…locally we’ve got something called Community Information Champions and I think a couple of our staff are trained, so a couple of receptionists, our healthcare assistant, they get additional training and normally it’s about accessing services, it’s our healthcare assistant she’s really good at that…”* [H8, GP, 17 Years of experience]
Knowledge	Knowledge/ lack of knowledge of support services (M)	*“I’d say good knowledge of what’s available but it’s probably not a very in-depth knowledge of what the service, potentially, will always offer.”* [H1, AHP, 5 Years of experience] “*I don’t have knowledge of what services are there.”* [H20, consultant, 22 Years of experience]
Memory, attention and decision processes	AHPs proactively searched for services to meet specific patient needs (E)	*“…the other day I saw a patient who might benefit from maybe like a befriending type scheme. So, I’m going to look into that for her… if I feel that there’s something a patient would benefit from, just come away and do my own kind of internet searching.”* [H16, AHP, 4 Years of experience]
**Patient factors**		
Social influences	Patients refusing referral to support services, denial, low education, IT illiteracy and comorbidities were barrier to addressing needs (B)	*“I think some people, not all, but they don’t really want that ongoing support. Cause obviously that’s a barrier in itself. Cause sometimes there’s patients where you feel that they would benefit more from it but if they’re not consenting then there’s nothing you can do.”* [H16, AHP, 4 Years of experience]
	Family members often supported patients to access services or online resources, and relayed/ repeated information (E)	*“Family are usually very good at helping. If family are available and around, they usually can be really good with directing or helping patients to work out what they need to access.”* [H1, AHP, 5 Years of experience]

**Fig. 1:**
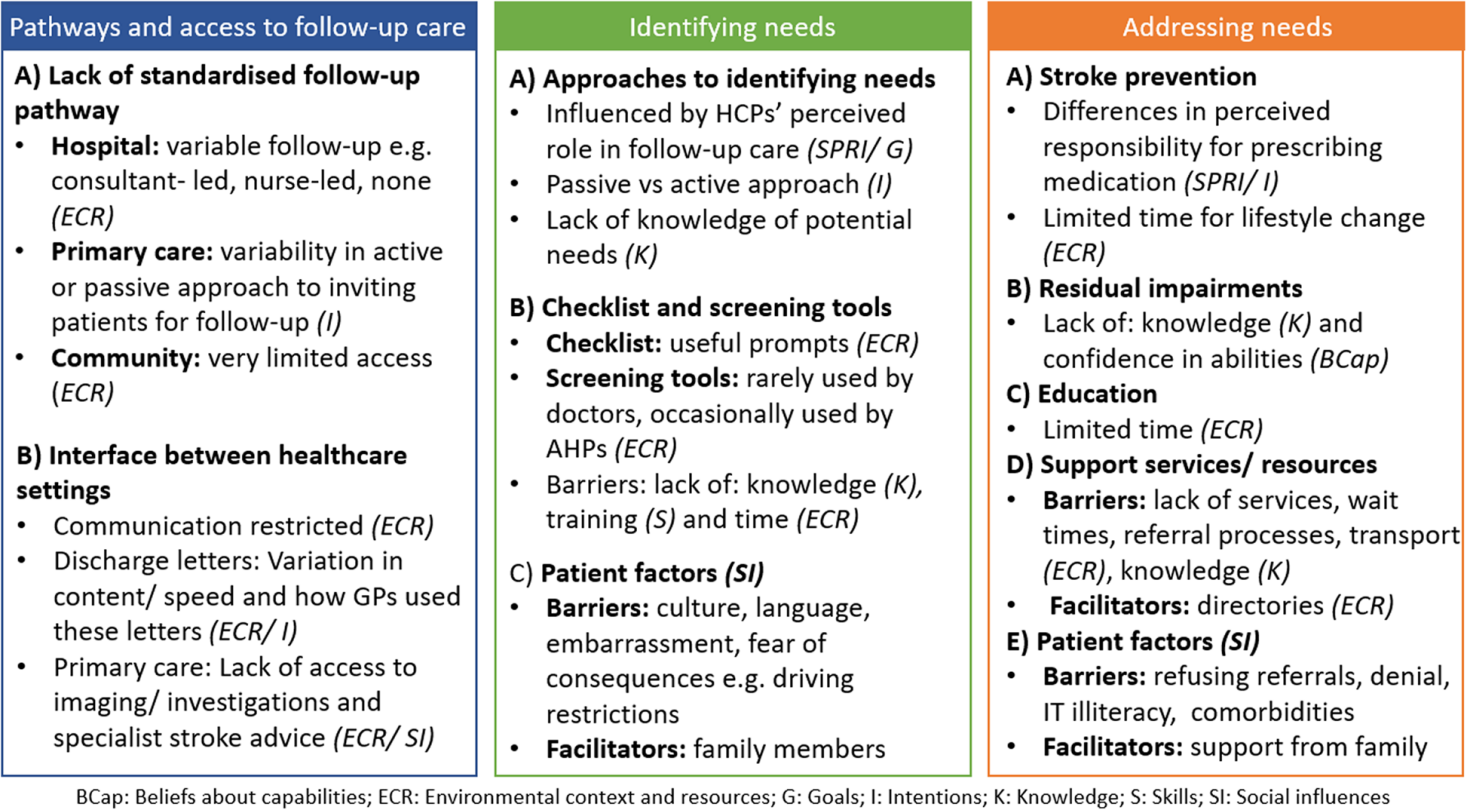
Summary of themes and subthemes

Themes and subthemes are described below and summarised in Tables 2 and 4 and Fig. 1.

### Pathways and access to follow-up care

#### Lack of standardised follow-up care pathway

Specialist review in secondary care is rapid with patients leaving hospital within hours or days. Subsequent follow-up is limited with no standardised follow-up care pathway and variability in follow-up between and within healthcare settings (Environmental context and resources). For example, one hospital had a nurse-led follow-up clinic, but consultants within that hospital differed in whether they referred their patients there or not (Intentions); whereas, another hospital had no follow-up (Environmental context and resources). The eligibility criteria to access early supported discharge varied across regions; minor stroke patients “rarely” received early supported discharge and TIA patients were not eligible (Environmental context and resources). In primary care, there was a spectrum of active vs passive approaches to follow-up with some general practices actively contacting patients and others relying on patients to contact them (Intentions). GPs often relied on annual long-term condition reviews as follow-up for these patients (Environmental context and resources).

#### Interface between healthcare settings

Communication between healthcare settings was restricted. It was usually one way and comprised electronic or paper discharge letters which varied in the speed they were sent, the content, and how the recipient HCP engaged with the letter (Environmental context and resources/ Intentions). Some GPs described frustration with the lack of access to results of imaging/ investigations and difficulty in contacting stroke consultants for specialist advice (Environmental context and resources/ Social influences).

### Identifying needs

#### Approaches to identifying needs

HCPs’ perceived role in follow-up care (Social professional role and identity) and knowledge of potential patients’ needs (Knowledge) influenced their approach to identifying needs. Consultants mostly considered the purpose of their follow-up clinics to be related to the acute management of TIA/ minor stroke, in particular interpreting and actioning imaging and investigations (Goal). For GPs, follow-up was predominantly focused on stroke prevention medication and risk factor management, such as blood pressure control (Goal). GPs and consultants were usually passive and relied on patients to raise issues themselves (Intentions/ Social influences). In contrast, nurses and AHPs took an active approach to identifying needs (Social professional role and identity/ Intentions). In general, nurses and AHPs had a more holistic understanding of potential needs and were confident in their ability to identify needs (Knowledge/ Beliefs about capabilities).

Overall, there was a lack of knowledge and understanding of sequelae post-TIA/ minor stroke, particularly amongst consultants and GPs who often viewed TIA/ minor stroke as transient events (Knowledge). Knowledge of residual impairments post-TIA/ minor stroke was gained through experience of following-up these patients (Knowledge) or, less commonly, through personal experience with family members (Social influences).

#### Use of checklists and screening tools

Checklists, usually developed in-house, were commonly used by nurses and AHPs and considered useful prompts (Environmental context and resources/ Beliefs about consequences). However, checklists were less commonly used by experienced nurses and AHPs who referred to using “instinct” to identify needs, gained through professional experience working in the field (Skill). In primary care, a long-term conditions template is used by nurses at annual reviews. However, the content was medically focused and influenced by a performance-based incentive: the Quality and Outcomes Framework (QOF) (Reinforcement).

AHPs varied considerably in their use of screening tools, if at all (Environmental context and resources). When used, screening tools informed decision making, such as psychology referral (Memory, attention and decision processes). In one early supported discharge team, the Barthel index was mandated by the local service commissioners (Reinforcement); however, this tool was not considered useful by AHPs (Beliefs about consequences). Screening tools were rarely used by GPs and consultants. Barriers to use of screening tools included lack of knowledge of available tools (Knowledge), lack of training to used and interpret tools (Skills) and lack of time (Environmental context and resources).

#### Patient factors

Culture and language were sometimes barriers to identifying needs (Social influences). There was a perception that patients do not want to raise non-medical or minor issues with doctors, but are more willing to discuss these problems with nurses and AHPs (Social influences). It was perceived that some patients withheld information due to embarrassment, not wanting to admit to problems for fear of consequences, such as driving restrictions (Social influences).

Family members facilitated identification of needs, in particular, women raised issues on behalf of their male partners (Social influences). On occasion, family members acted as translators (Social influences). However, in some instances, presence of family could be a barrier if patients were embarrassed to discuss problems in front of them (Social influences).

### Addressing needs

#### Stroke prevention

There was disparity between consultants and GPs regarding perceived responsibility for prescribing stroke prevention medication (Social professional role and identity). Some consultants considered this to be part of their role (Social professional role and identity), but varied in tailored versus protocolised approaches to prescribing (Intentions). In contrast, other consultants were less confident prescribers and relied on GPs (Beliefs about capabilities/ Intentions). Some GPs perceived preventative medicine as a strength of general practice and believed prescribing medication was their role, particularly in the context of comorbidities and polypharmacy (Social professional role and identity/ Goal). However, other GPs felt they lacked specialist stroke knowledge and relied on stroke consultants (Belief about capabilities/ Social influences). This influenced whether GPs actively saw patients and reviewed their medication or simply issued repeat prescriptions based on secondary care recommendations (Intentions).

Although HCPs recognised the importance of lifestyle change for stroke prevention (Beliefs about consequences), this was rarely addressed in a meaningful way or actively supported (Intentions). Time constraints were a key barrier (Environmental context and resources); however, behaviours were also influenced by lifestyle change not being considered a key goal of their follow-up (Goal/ Social professional role and identity) and lack of knowledge of support services and resources to support lifestyle change (Knowledge). As a substitute, HCPs used information leaflets and consultants made recommendations to GPs through discharge letters (Environmental context and resources).

#### Residual impairments

Consultants rarely addressed residual problems, such as fatigue, psychological and cognitive problems, due to lack of knowledge of potential problems and lack of confidence in how to address these problems (Knowledge/ Beliefs about capabilities). Although most GPs lacked understanding of residual problems related to TIA and minor stroke, they were aware of holistic consequences from long-term conditions in general (Knowledge); however, restricted time (typically within 10-min appointments) and prioritisation of stroke prevention – partly influenced by performance based financial incentives (QOF) – were commonly cited as reasons for not addressing these issues (Environmental context and resources/ Intentions/ Reinforcement).

AHPs and nurses considered addressing holistic needs part their role and were confident in their ability to address residual needs (Social professional role and identity/ Beliefs about capabilities). Often AHPs and nurses had the skills to actively addressed residual needs, such fatigue management, and tailored their approach to patients’ demographic traits and personal circumstances (Skill/ Memory attention and decision processes). Many AHPs and nurses also believed in the value of a “supportive chat” which involved active listening, acknowledging patients’ needs and reassurance (Beliefs about consequences) and recognised the importance of lay language (Skill).

#### Education about diagnosis, stroke risk and medication

Consultants usually provided information on diagnosis and stroke risk (Intentions); however, this was not comprehensive and there was belief that it is difficult for patients to retain information provided at the acute stage (Beliefs about consequences). Most GPs did not routinely provide education due to time constraints and an assumption that this had been done in secondary care (Environmental context and resources/ Social professional role and identity). Most nurses and AHPs actively provided education and considered this part of their role (Intentions/ Social professional role and identity). They often used stroke charity websites to supplement verbal information (Environmental context and resources).

#### Use of support services and resources

Support services were mentioned as potential sources of support, both formal healthcare services, such as smoking cessation and talking therapy, and local community groups (Environmental context and resources). However, barriers to accessing services were reported, including a lack of services, long wait times to access services, lengthy or complicated referral processes, transport issues, and geographical boundaries (Environmental context and resources).

Another important barrier was lack of knowledge of services, particularly as they changed rapidly (Knowledge). In-house directories were sometimes used in primary and community care; however, directories were often outdated unless someone was delegated to regularly update them (Environmental context and resources). In early supported discharge teams, knowledge of support services was facilitated by multidisciplinary team working, building relationships in the community and experience of working in the area (Knowledge). AHPs also proactively searched for services to meet specific patient needs (Memory, attention and decision processes). In primary care, GPs having access to social prescribers or community champions facilitated identification of support services (Environmental context and resources).

#### Patient factors

Patient factors were sometimes barriers to addressing needs, including patients refusing referral to support services, denial, low education, IT illiteracy and comorbidities (Social influences). Family members were often facilitators and supported patients to find or access services, access the internet or computers, relaying or repeating information (Social influences).

## Discussion

There is no clear pathway for supporting people with TIA or minor stroke after rapid specialist review in hospital. Consequently, these patients have limited access to HCPs from all settings. HCPs’ approaches to identifying and addressing needs are predominantly influenced by their professional training, perceived role and purpose of their clinic, knowledge of potential needs, and time constraints. In general, consultants and GPs are medically focused; whereas, nurses and AHPs are more holistic in their approach to follow-up care. However, TIA/ minor stroke patients are unlikely to receive follow-up from nurses and AHPs.

### Comparison with other studies

This is the first study to explore perceived influences on HCPs’ behaviours related to follow-up care post-TIA/minor stroke, specifically identification and management of patients’ needs. In stroke research, HCPs’ perceived roles and beliefs about consequences have been found to influence rehabilitation assessment and referral practices on Australian acute stroke units [19]. Similarly, in primary care, research shows disparity in GPs’ perception of their role in cardiovascular disease prevention, in particular lifestyle advice, and patient factors were important influences on GPs actions [20, 21]. We identified sub-optimal and variable interface communication between healthcare settings, which is supported by other stroke research [22]..

Lack of adequate follow-up is reflected in studies exploring perspectives of follow up from people with TIA and minor stroke, which found these individuals feel abandoned post-discharge [5]; frustrated due to lack of recognition of problems from HCP [23]; and dissatisfied with the lack of communication [24], holistic care [25], rehabilitation options [10], and individualised information and support [24]. Knowledge of stroke and medication has been identified as a barrier to secondary stroke prevention medication adherence; therefore, inadequate follow-up care adversely affects both patients satisfaction with care and clinical outcomes [26]..

### Implications for clinicians and policy makers

Research has shown that structured stroke care at the post-acute stage is essential to improve secondary prevention post-stroke [27–30]. However, holistic care should also incorporate management of residual problems and education about diagnosis and stroke risk. There is no clear follow-up pathway to support people after TIA/ minor stroke and HCPs have disparate views on their responsibility and role in follow-up, which may mean patients slip through the net and receive no follow-up. We have identified factors that influence HCPs actions related to follow-up care post-TIA/ minor stroke which can help inform guidelines to improve healthcare for these patients. A key recommendation is to develop a structured follow-up pathway which incorporates specialist stroke care from consultants, holistic care from stroke nurses/ AHPs and long-term care from primary care GPs/ nurses. HCPs roles within this pathway should be clearly defined (Social professional role and identity) to influence their Intentions and Goals.

### Future research

Identification and management of needs post-TIA/minor stroke is currently sub-optimal. Through identifying TDF domains relevant these behaviours, our findings provide an understanding of what needs to change to improve follow-up post-TIA/minor stroke and potential intervention targets. Future research should use this knowledge to inform intervention development. The Behaviour Change Wheel [31], a framework for behaviour change intervention design, can be used to systematically link the relevant TDF domains to ‘intervention functions’. For example, the TDF domain ‘knowledge’ (lack of knowledge of TIA/minor stroke patients’ needs) maps to the intervention function ‘education’. Multiple intervention functions can be included in a complex intervention to change HCPs’ behaviour. Previous follow-up interventions post-TIA/minor stroke have focused on secondary prevention medication and lifestyle change [28]; however, it is important for future interventions to address holistic needs [5].

### Strengths and limitations

A strength of our study is that we drew upon a wide range of HCPs experiences across different healthcare settings and disciplines. These different perspectives enriched our understanding of the diverse range factors influencing follow-up care across different settings. Through use of TDF, we used behavioural theory to identify influences on behaviour (i.e. identifying and addressing needs post-TIA/ minor stroke), which are potentially amenable to change and could be targeted to improve care. An advantage of TDF is that it links to the Behaviour Change Wheel which provides a methodology for progressing to developing behaviour change intervention [17]. However, a limitation of collecting TDF data though interview is that we can only describe participants’ reported influences on care after TIA and minor stroke and such perceptions do not necessarily fully reflect actual influences. A limitation of our study is that, for pragmatic reasons, only a subset of transcripts were double coded. Although it was not the purpose of our study to be generalisable, we only sampled participants from the West Midlands. Therefore, future research could explore if our findings are consistent across other geographical locations.

## Conclusion

Following rapid discharge from hospital, people with TIA or minor stroke have limited access to follow-up care and care they do receive is inadequate. Through identifying factors which influence follow-up care of these patients, we can inform guidelines and practical strategies to improve holistic healthcare post-TIA and minor stroke.

## Supplementary Information

**Additional file 1: eTable 1**. Theoretical Domains Framework (TDF) version 2. **eTable 2**. Characteristics of healthcare provider participants.

## Data Availability

The datasets used and/or analysed during the current study available from the corresponding author on reasonable request.

## Abbreviations

AHP: Allied health professionals
GPs: General practitioners
HCP: Healthcare providers
TDF: Theoretical Domains Framework
TIA: Transient ischaemic attack
