# Supplementary material for: Factors influencing follow-up care post-TIA and minor stroke: a qualitative study using the theoretical domains framework
Source: BMC Health Serv Res. 2022 Feb 21;22:235. doi: 10.1186/s12913-022-07607-0 (PMC8859903; doi:10.1186/s12913-022-07607-0)
Supplement: Supplementary file 1 — Additional file 1: eTable 1. Theoretical Domains Framework (TDF) version 2. eTable 2. Characteristics of healthcare provider participants. [file 12913_2022_7607_MOESM1_ESM.docx]

**eTable 1: Theoretical Domains Framework (TDF)** **version 2**

| **Domain** | **Definition** |
| --- | --- |
| 1. Knowledge | An awareness of the existence of something |
| 2. Skills | An ability or proficiency acquired through practice |
| 3. Social/professional role and identity | A coherent set of behaviours and displayed personal qualities of an individual in a social or work setting |
| 4. Beliefs about capabilities | Acceptance of the truth, reality or validity about an ability, talent or facility that a person can put to constructive use |
| 5. Optimism | The confidence that things will happen for the best or that desired goals will be attained |
| 6. Beliefs about Consequences | Acceptance of the truth, reality, or validity about outcomes of a behaviour in a given situation |
| 7. Reinforcement | Increasing the probability of a response by arranging a dependent relationship, or contingency, between the response and a given stimulus |
| 8. Intentions | A conscious decision to perform a behaviour or a resolve to act in a certain way |
| 9. Goals | Mental representations of outcomes or end states that an individual wants to achieve |
| 10. Memory, attention and decision processes | The ability to retain information, focus selectively on aspects of the environment and choose between two or more alternatives |
| 11. Environmental context and resources | Any circumstance of a person’s situation or environment that discourages or encourages the development of skills and abilities, independence, social competence and adaptive behaviour |
| 12. Social influences | Those interpersonal processes that can cause individuals to change their thoughts, feelings, or behaviours |
| 13. Emotion | A complex reaction pattern, involving experiential, behavioural, and physiological elements, by which the individual attempts to deal with a personally significant matter or event |
| 14. Behavioural regulation | Anything aimed at managing or changing objectively observed or measured actions |

*Atkins L, Francis J, Islam R, O’Connor D, Patey A, Ivers N, et al. A guide to using the Theoretical Domains Framework of behaviour change to investigate implementation problems. Implementation Science. 2017;12(1):77.*

**eTable 2: Characteristics of healthcare provider participants**

| **ID** | **Age (years)** | **Sex** | **Profession** | **Healthcare setting** | **Years of experience** |
| --- | --- | --- | --- | --- | --- |
| H1 | 21-30 | Female | AHP (Physio) | Community care | 5 |
| H2 | 41-50 | Female | AHP (SLT) | Community care | 3 |
| H3 | 41-50 | Female | AHP (Physio) | Secondary & community care | 23 |
| H4 | 51-60 | Female | Nurse | Community care | 37 |
| H5 | 41-50 | Female | AHP (OT) | Community care | 16 |
| H6 | 41-50 | Female | AHP (Physio) | Community care | 18 |
| H7 | 51-60 | Male | Stroke consultant | Secondary care | 20 |
| H8 | 31-40 | Male | GP | Primary care | 17 |
| H9 | 31-40 | Male | GP | Primary care | 6 |
| H10 | 51-60 | Male | GP | Primary care | 31 |
| H11 | 41-50 | Male | GP | Primary care | 18 |
| H12 | 31-40 | Male | GP | Primary care | 7 |
| H13 | 31-40 | Male | GP | Primary care | 13 |
| H14 | 31-40 | Female | AHP (OT) | Community care | 17 |
| H15 | 41-50 | Female | AHP (Psychologist) | Secondary & community care | 4 |
| H16 | 31-40 | Female | AHP (Physio) | Secondary & community care | 4 |
| H17 | 41-50 | Female | Stroke consultant | Secondary care | 8 |
| H18 | 41-50 | Female | AHP (Psychologist) | Secondary care | 12 |
| H19 | 41-50 | Female | Nurse | Secondary care | 12 |
| H20 | 41-50 | Male | Stroke consultant | Secondary care | 22 |
| H21 | 41-50 | Male | Stroke consultant | Secondary care | 24 |
| H22 | 31-40 | Female | Nurse | Secondary care | 13 |
| H23 | 31-40 | Female | Nurse | Secondary care | 10 |
| H24 | 41-50 | Male | Stroke consultant | Secondary care | 12 |
| AHP: Allied Health Professional; OT: Occupational Therapist; Physio: Physiotherapist; SLT: Speech and Language Therapist | | | | | |
